# Domestic Waste Classification Behavior and Its Deviation from Willingness: Evidence from a Random Household Survey in Beijing

**DOI:** 10.3390/ijerph192214718

**Published:** 2022-11-09

**Authors:** Ben Ma, Yixuan Jiang

**Affiliations:** School of Environment and Natural Resources, Renmin University of China, Beijing 100872, China

**Keywords:** waste classification willingness, waste classification behavior, deviation between willingness and behavior, ordered logit model, Beijing

## Abstract

To realize widespread domestic waste classification (DWC), deviation between residents’ waste classification willingness (WCW) and waste classification behavior (WCB) needs to be reduced. Based on an extended framework of the theory of planned behavior, this study investigates residents’ WCW, WCB and their deviation through a random face-to-face survey of 632 households in Beijing. By employing the ordered logit model, determinants have been empirically revealed. We find that there is a deviation between WCW and WCB. 54.3% of respondents reported a higher degree of WCW than that of WCB. The deviation is determined by specific external conditions, including attitudes and norms associated with other subjects that are not authoritative regulators, and the implementation of various policy instruments for DWC. The higher the satisfaction with the services of property enterprises, the higher the deviation is. The positive WCB of peers in communities failed to create positive subjective norms, but instead exacerbated free-rider phenomenon by inducing their negative WCB. Flat-rate charge, publicity measures and the effectiveness of DWC within community can significantly reduce the deviation. On this basis, policy suggestions are put forward to further reduce the deviation and improve the rate of DWC.

## 1. Introduction

With the acceleration of urbanization and industrialization and the rapid increase in the urban population, the massive generation and disposal of domestic solid waste have become a major challenge for urban sustainable development [1,2]. It presents a clear trend for countries worldwide to promote source domestic waste classification (DWC), which is an essential way to realize separated waste for tailored safe disposal, resource recovery, and to reduce the total cost of waste disposal for the whole society [3,4,5]. Many developed economies have implemented sustainable municipal solid waste management through strict legislation and policies to separate different kinds of refuse within households, reuse scraps, and recycle valuable resources found in domestic solid waste [6]. As a fast-developing country, China started exploration of DWC during the 1990s. In 2000, eight large cities, including Beijing and Shanghai, were the first batch of cities to carry out a DWC pilot across China [7]. However, most of the pilot projects were not successful primarily due to a relatively low level of public engagement and a limited scope in terms of the area of implementation [8]. Meanwhile, residents in some cities had a high willingness to separate waste but did not actually do so. Thus, there was a deviation between willingness and behavior of waste classification [9]. In 2017, China announced a new expanding policy by selecting 46 cities, including megacities like Beijing and Shanghai, to carry out another round of pilot projects for comprehensive DWC [10]. Given that the deviation still existed [1], it is urgent and necessary to narrow the gap between willingness and behavior of waste classification [11]. Because DWC within households involves every resident’s behavior, the effective management of comprehensive DWC is a resource-intensive issue. Accordingly, public policy intervention is essential to help promote habit formation of DWC at source, where it is a major test for grassroots governance capacity. Beijing, the large capital city of China, is under urgent pressure to reduce domestic solid waste by household classification and resource recycling. The city has been included twice in national designated pilots for waste classification. Along with the promulgation of the updated Beijing Decree on Domestic Waste Management in 2020, the policy of comprehensive DWC began to be faithfully implemented (According to the Beijing Municipal Commission of Urban Management, Beijing has made significant progress in DWC in recent years, with residents’ awareness rate and participation rate of DWC increasing to 98% and 90% respectively by April 2021. However, field investigation of this study found that secondary sorting in front of the dumpster stations by volunteers or employees of property management enterprises is popular. Thus, the actual DWC within households still presents significant potential to be further improved. Regarding the policy implementation, the deviation between residents’ willingness and behavior is worth further examination). As residents are the ultimate implementer of DWC, their willingness of waste classification becomes an important determinant for residents to carry out waste classification task to perform the anticipated effect at the city level. In this context, investigating residents’ source waste classification willingness (WCW) and waste classification behavior (WCB), and then analyzing the deviation between WCW and WCB and its determinants by taking Beijing as the case, are of great policy significance for optimizing public interventions to promote a comprehensive development of the environment-friendly habit by establishing a long-effect mechanism for source waste separation.

DWC implies change of willingness and behavior as the starting point and thus heavily relies on household and individual participation [6]. The focus of scholars has gradually shifted from municipal solid waste disposal to analyzing residents’ domestic garbage separation willingness and behavior and their underlying mechanisms [12]. The current research concentrates on the following aspects: first, it explores WCW or WCB of residents and their influencing factors separately through experiments or field survey [13,14]; second, it discusses the relationship between WCW or WCB and their determinants based on the theory of planned behavior, attitude-behavior-condition (A-B-C) theory and others [2,11,15,16], and a positive correlation has been found between willingness and behavior of waste classification; third, it finds out and analyzes the deviation between WCW and WCB, and attempts to explain the reasons for the deviation by comparing different impacts of the same factors on WCW and WCB [1,9,15]. However, these studies have failed to build specific variables for the deviation between willingness and behavior, and they have not conducted in-depth quantitative studies by targeting cities that have implemented waste classification policies comprehensively. Therefore, the research on the extent and determinants of deviation between residents’ WCW and WCB needs to be better understood via further investigation.

Due to the general reality that there are deviations between residents’ waste classification willingness and behavior in many cities and the fact that the sources of deviations in the existing literatures need to be further explored, this study conducted a face-to-face random questionnaire survey to investigate the deviation between residents’ WCW and WCB after the implementation of comprehensive waste classification policies in Beijing, and analyzed the determinants of the deviation through an extended framework of the theory of planned behavior incorporating policy implementation factors. Then, this paper accordingly evaluated the effectiveness of the implementation of existing regulatory, incentive, and publicity policies, and proposed corresponding policy recommendations to promote the transformation from residents’ WCW into WCB.

This paper has several novel aspects. First, this paper constructs a self-contained scenario framework to measure residents’ waste classification willingness and behavior by including all kinds of domestic waste for constructing DWC indicators, which is more realistic in modeling WCW, WCB, and their deviation. According to Beijing’s DWC policy, four categories of domestic waste, including recyclables, food waste, hazardous waste, and other waste, are used to construct classification variables, which is more comprehensive than a binary classification of 0–1 variables (YES or NO). Second, this paper updated the assessment method of deviation between residents’ waste classification willingness and behavior. We not only horizontally compares the differences between influencing factors of WCW and WCB but builds a specific variable for their deviation degree by combining willingness and behavior. In this way, an induced mechanism of deviation generation or reduction can be revealed more directly and accurately. Third, this paper adding different categories of policy factors to the traditional theoretical framework of planned behavior, such as regulation, incentive, and publicity, to fully consider the impact of established policies on deviations between WCW and WCB, whereby the conclusions drawn are of more practical value for policymaking in the context of comprehensive domestic waste classification policy implementation.

The rest of the paper is organized as follows. Section 2 presents the theoretical basis and literature review. Section 3 introduces the case selection, research design, and household survey process. Section 4 presents the variable construction, model specification, and descriptive statistics. The empirical results are reported and discussed in Section 5. Section 6 provides the main conclusions and policy implications.

## 2. Theoretical Basis and Literature Review

On the relationship between DWC willingness and behavior, scholars have two research focus. The first one is that willingness can guide the actual occurrence of behavior, and these two aspects present certain consistency. The theory of planned behavior believes that human behavior is a reflection of willingness, which is influenced by attitudes, subjective norms, and perceived behavioral control. The perceived behavioral control may act directly on behavior not through willingness [17]. Thus, in most cases, residents’ WCW can be used as a direct predictor for WCB. Findings by Tonglet et al. [18], Miafodzyeva et al. [19], and Fan et al. [2] have confirmed that residents’ WCW is the most effective predictor of WCB, with a significant positive correlation between them. The second view is that individual behavior is not fully determined by willingness, and willingness cannot be completely translated into behavior, thus leaving a gap between them. Czajkowski et al. [20] found that residents always expressed a relatively strong WCW, but their actual WCB was typically lower. Nevertheless, external conditions such as waste-sorting infrastructure, government publicity, and incentives can moderate the relationship between residents’ waste classification willingness and behavior [15] and convert willingness into actual behavior [21].

Waste classification research in China has also revealed that residents’ higher WCW did not necessarily result in corresponding higher WCB. Chen et al. [9] found in a survey data of residents in six districts of Ningbo that there was a discrepancy between residents’ waste classification willingness and behavior, with urban residents having a substantially higher proportion of WCW (82.5%) than actual WCB (13%). Kuang and Lin [1] conducted a survey based on public participation in DWC in Beijing, Shanghai, Guangzhou, and Shenzhen in 2019. They reported that 99.1% of respondents in Beijing were willing to sort domestic waste, whereas only 82.4% of respondents did so in their daily routines. Both the above two researches constructed binary variables for WCW and WCB. Wang [11] divided behavioral willingness into target-based and perform-based and found that both could only explain 15% of the discrepancy in residents’ waste classification behavior, suggesting that a large gap exists between residents’ willingness and actual behavior. The analysis also found that the effectiveness of waste-sorting policies could adjust the impact of target-based willingness on behavior and might be one of the reasons for the deviation between WCW and WCB.

Among the factors influencing residents’ DWC, willingness and behavior are the key research topics. Residents’ WCW and actual WCB can be used as representations of residents’ waste classification status [2,14,22]. Scholars typically use the theory of planned behavior (TPB) [17], attitude-behavior-condition (A-B-C) theory [23] and other theories to analyze the factors influencing residents’ waste classification willingness and behavior. TPB assumes that human behavior is the result of deliberate planning. It contains five variables: attitude, subjective norms, perceived behavioral control, intention (Ajzen [17] defined the behavioral intention in the rational behavior theory as follows. Intention refers to the thought tendency and action motivation before taking action. "Waste classification willingness" in this paper refers to the intention of residents to actually separate waste in the context of existing waste classification policies in Beijing. Thus, willingness in this paper can be equated with the concept of behavioral intention proposed by Azjen), and behavior. As a classic theory to explain individual’s behavior, TPB has been widely used in the field of household waste management. However, many scholars, including Ajzen, believe that when there is a large deviation between intention and behavior, additional variables should be included based on the original three kinds of variables of attitude, subjective norm, and perceived behavioral control to improve the explanatory power of the model [15]. The A-B-C theory proposed by Guagnano et al. [23] for predicting environmental behavior, holds that environmental behavior is determined by environmental attitudes and external conditions. Policy factors are thus added as common external condition factors to extend TPB model [15,16,24]. The extended framework of TPB in this thread of literature includes the following five aspects: (1) Attitudes, including environmental consciousness [1], trust [25], knowledge of waste classification [9], and attitudes toward DWC are generally positively related to waste classification willingness and behavior. Heterogeneities may emerge due to different situation of regional cultural education [19]. (2) Subjective norms, including the social pressure from the behavior of peers or groups to create psychological conformity are also examined [14,26]. This effect is usually positive, but it may also lead to “free-riding” in the absence of supervision. The negative effect is still lacking empirical evidence. (3) Perceived behavioral control denoting the level of difficulty regarding waste classification as perceived and assessed by residents, which is mainly influenced by past sorting experience, time cost, and the accessibility of waste-sorting facilities [18,22,27], is addressed. (4) Policy intervention factors, including laws and regulations [19,25], incentives [20,28,29], publicity measures [3,30], or a general consideration of whether surveyed respondents are in a pilot area for waste classification are included [1,9]. (5) Sociodemographic characteristics, including gender, age, education level, income, etc., of which the sign, symbol, and significance of their effects vary greatly among different survey regions and groups, are addressed [1,9,31].

To sum up, studies on waste classification willingness and behavior of urban residents presents the following characteristics. First, in terms of research objects, the investigation in China primarily focused on the first round of pilot cities, whose implementation effects of DWC were generally unsatisfactory during the pilot phase. The studies concerning the new round of pilot cities for waste classification are still insufficient [1], especially for cities such as Beijing, where comprehensive waste classification is carried out by introducing a package of policies. There is still a research gap concerning an in-depth examination of those measures and their influences on the deviations between residents’ WCW and WCB [15,29]. Second, existing studies only construct binary variables to analyze the influencing factors of WCW and WCB separately, and the deviation between WCW and WCB still lacks specific and faithful measurement and in-depth exploration [1,9]. The rough definition of waste classification variables cannot match the four-type standard of DWC defined by the policy in Beijing, the different strength of WCW, and the multiplicity feature of WCB. It is also insufficient to explain the impact mechanism and impact degree for the deviations though analyzing the determinants of willingness and behavior separately. Therefore, the factors influencing the deviations between willingness and behavior of waste classification deserve to be further explored.

## 3. Research Design and Household Survey

### 3.1. The Case of Beijing

As the capital city, Beijing is one of the first in China to carry out DWC. The pilot for DWC dates back to 2000, but the first round of waste classification did not achieve significant results (According to the 2011 Beijing Municipal Household Waste Report released by the Friends of Nature, only residents in 4.4% of communities have achieved standard DWC. In 41.1% of communities, residents’ waste classification behavior has not changed. In the remaining 50% of communities, only a few residents sort their garbage, and residents’ participation in source DWC is insufficient as the habit of DWC has not been developed). Since the implementation of the newly revised Decree on Domestic Waste Management on 1 May 2020, Beijing has entered a new stage of comprehensive implementation of DWC. After more than a year of efforts, Beijing has realized remarkable outcomes in its waste classification management system, policy intervention with mixed tools, and general effectiveness of DWC.

Beijing has established a unique management system for DWC. First, the legal status of DWC is established and highlighted through the highest ranking of local legislation. Second, the city government has set up a DWC promotion headquarters headed by city leaders, and Beijing Municipal Commission of Urban Management is responsible for the waste classification administration. The urban management department of each district, each street administrative office, and the community residential committee are responsible for the management of DWC within their jurisdictions. At the same time, the legislation specifies that property enterprises in communities are responsible for the concrete management of the neighborhood residents’ DWC.

Beijing has adopted command-and-control, economic-incentive, and information-providing policies to improve residents’ WCW and WCB: (1) Command-and-control policies. Governments conduct top-down performance assessments toward public authorities and impose inspection and punishment to community property enterprises. Simultaneously, the main responsibility of individual waste classification is clearly defined that those who fail to sort their domestic waste according to the classification standard will be educated and dissuaded, and those who refuse to correct themselves will be asked to participate in community service or be issued a fine. (2) Economic-incentive policies. Beijing formulated and released flat charge policy for domestic waste disposal (According to the document No. 68 of Beijing Government Office [1999], Beijing has levied urban domestic waste disposal fee since September 1999. The rate is 3 RMB/month for each household of the city residents and 2 RMB/month for each flowing person in Beijing) and domestic waste clearance (According to the Beijing Price (Collection) Document [1999] No. 253, the domestic waste clearance fee of annual 30 RMB/household is charged to the owners of houses within the city) [32]. The charging rate aggregates to 66 yuan per year, which is a fixed charge introduced in 1999. According to field survey, the collection rate of domestic waste clearance fees is much higher than the disposal one because the latter is imposed by township governments with higher levy costs than that by community property enterprises together with other routine service fees. Besides, a fraction of communities implement points exchange systems for waste classification by providing honors and awards. (3) Information-providing policies. Large-scale publicity and education programs have been carried out, including the establishment of a column on the government portal, the organization of lecture groups, precise door-knocking actions, and publicity and guidance in parks and subway stations. Diversified contact and non-contact publicity modes are adopted in each community according to its own characteristics.

**Figure 1:**
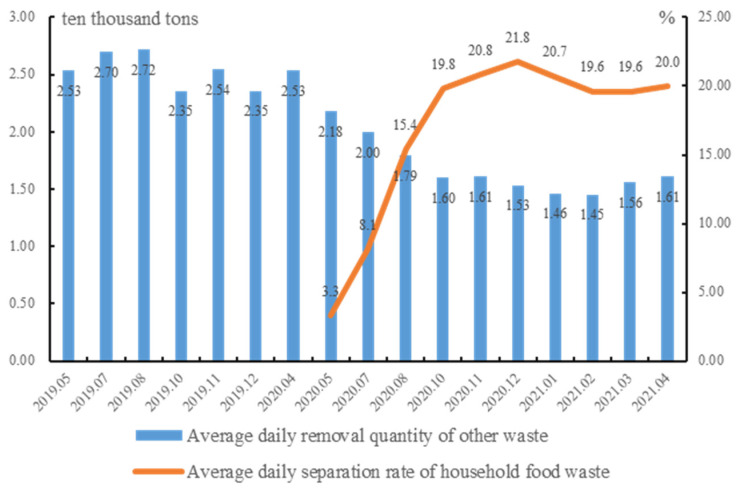
Average daily household food waste separation rate and other waste removal quantity in Beijing. Data source: Press conferences on the implementation of the 2020 Beijing Decree on Domestic Waste Management.

Beijing has achieved great progress in DWC. The total domestic waste removal volume has decreased significantly, with an average daily removal volume of 20,600 tons from January to April 2021, dropping by 25.6% compared to that of 2019. Furthermore, the amount of separated recyclables and the recycling rate have improved. The recycling rate of domestic waste stabilized at about 35% by April 2021, an increase of 7.6% compared with the average value in 2020. The amount and rate of separated food waste have also increased significantly. The separation quantity reached 4248 tons per day in December 2020, 13.7 times that of April 2020. The separation rate reached 21.78% in December 2020. In addition, the amount of other waste has decreased significantly. The average daily removal quantity in May 2020 was 21,800 tons per day, a reduction of 14% from 2019. This figure further dropped to 15,300 tons per day in 2020, presenting year-on-year decreases of nearly 35% (Figure 1).

Generally, the habit development of comprehensive waste classification will be a gradual and long-term process. In Beijing, it is still a common phenomenon to observe non-universal and incomplete source DWC. The conducted field survey indicates that waste classification in Beijing still relies heavily on the secondary sorting by property enterprises’ staff on duty in front of public waste bins within communities. Therefore, it is relevant to analyze how much deviation there is in residents’ waste classification willingness and behavior in the context of implementation of comprehensive DWC policies and what factors determine the deviation in order to better understand and profoundly improve waste classification management and policies in Beijing.

### 3.2. Research Design

**Figure 2:**
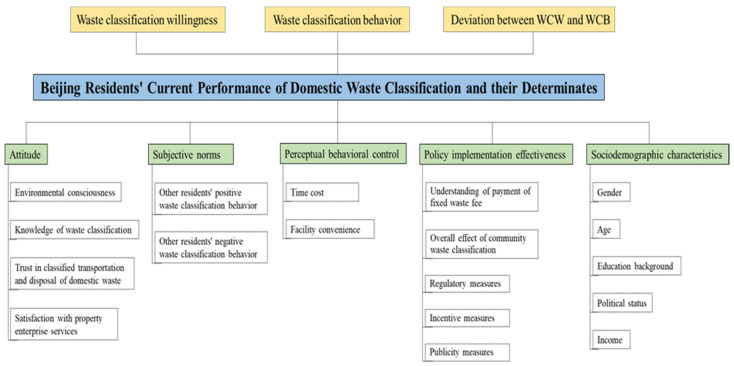
Research framework of residents’ WCW, WCB and their deviation.

This study analyzes residents’ WCW and WCB by focusing on their deviation. A research framework is established based on the theory of planned behavior [17] and adds two types of variables, policy measures and sociodemographic characteristics. In this way, the effect of five factors on WCW, WCB, and their deviation can be further explored, including attitude, subjective norms, perceived behavioral control, policy implementation effectiveness, and sociodemographic characteristics. These factors are specified into 18 sub-categories, and the research framework is shown in Figure 2: specifically, (1) attitude, including environmental consciousness, knowledge of waste classification, trust in classified transportation and disposal of domestic waste, and satisfaction with property enterprise services; (2) subjective norms, focusing on peer effects of residents’ both positive and negative waste classification behavior; (3) perceptual behavioral control, including time cost and facility convenience; (4) policy implementation effectiveness, including the related fixed waste fee implemented since 1999, the overall outcome of community waste classification after the new round of DWC pilot, and the regulatory measures, incentive measures, and publicity measures implemented in the community during the pilot; (5) sociodemographic characteristics, including gender, age, education background, political status, and income.

This study designed the questionnaire to include three parts with 18 multiple-choice questions and 2 fill-in-the-blank ones. The first part asks about the current status of residential waste classification, including individuals’ willingness and actual behavior regarding DWC. The second part investigates the factors that affect WCW and WCB. The third part obtains respondents’ sociodemographic characteristics, including age, gender, educational background, political status, and income. Through presurvey, the questions were improved one by one according to their suitability, so that the final questions are more accurate, fit better with residents’ daily life and improve the reliability and accuracy of responses.

### 3.3. Household Survey

Two of the six main urban districts in Beijing were randomly selected for the survey. The Fengtai and Chaoyang districts were finally targeted. Then, three residential communities with property service enterprise were selected for random surveys in the two districts respectively. Residents in Zfy communities I, II, and III in Fengtai District and Zyc communities A, B, and C in Chaoyang District comprised the population. Specifically, the geographic location of the research sites is representative. The selected communities include two groups of neighborhoods, one in the south and the other in the north of Beijing. These two groups belong to two administrative districts, which can reflect variance in the DWC of different areas in Beijing. Secondly, the age of the buildings is representative. The Zfy communities were put into use between 1997 and 2007 and the Zyc neighborhood after 2010, representing typical commercial housing of different ages. Third, the type of living is representative. The average proportion of those renting in the six communities is about one quarter, covering the two main types of living, including owner occupied and renting. Lastly, the current status of DWC is representative. These communities have a relatively sound data base and full participation of the government, communities, and residents in the implementation of waste classification policies.

A total of 4026 households in the six communities were randomly sampled by door number information using a 20% sampling ratio. After professional training for the investigators, the questionnaire survey was conducted in a face-to-face manner. The investigators provided necessary preset objective interpretation for questions to avoid response errors caused by respondents’ misunderstanding. A total of 632 valid questionnaires were obtained, excluding cases such as refusal to visit and long-term unattended households. After cleaning the data, the descriptive statistics of the basic sample characteristics are shown in Table 1. Sample characteristics such as gender, age, and education level are balanced or consistent with main participants in DWC in Beijing. The proportion (46.27%) is the highest for personal annual income ranging from 30,000 to 100,000 yuan. This is basically consistent with a per capita disposable income for urban residents of 75,600 yuan in 2020 in Beijing.

## 4. Model Specification and Descriptive Statistics

### 4.1. Variable Construction

The definitions and descriptive statistics of the variables constructed in this paper are reported in Table 2. The dependent variables include the following three items: waste classification willingness (WCW), waste classification behavior (WCB), and deviation variables based on the interaction of the two. The independent variables include five major categories and 18 variables, most of which are categorical variables. Time cost and income variables are continuous ones.

### 4.2. Model Specification

The dependent variables are categorical ones, and their numerical values correspond to the degree level in sequence. In other words, the dependent variables are inherently ordinal. So, the ordered logit model is proposed for regression analysis [33]. In order to analyze the factors determining residents’ WCW, WCB, and DWB (abbreviation for the degree of deviation between WCW and WCB), the model is specified as follows:

(1) $$\;Y_{i}^{*}=\beta_{0}+\beta_{1}X_{1i}+\beta_{2}X_{2i}+\beta_{3}X_{3i}+\ldots +\beta_{17}X_{17i}+\beta_{18}X_{18i}+\varepsilon_{i}$$

In the above equation, $Y_{i}\;$=$\;Y_{WCW}$ or $Y_{WCB}$ or $Y_{DWB}$, $\beta_{0}$ is the constant term, $\beta_{1}$–$\beta_{18}$ are the parameters to be estimated, and ε is the random error term.

(2) $$Y_{WCW}=\{\begin{array}{c} 1,\;\;\;\;\;Y_{WCW}^{*}<\mu_{1}\\ 2,\;\;\;\;\;\;\mu_{1}<Y_{WCW}^{*}<\mu_{2}\\ 3,\;\;\;\;\;\;\mu_{2}<Y_{WCW}^{*}<\mu_{3}\\ 4,\;\;\;\;\;\;\mu_{3}<Y_{WCW}^{*}<\mu_{4}\\ 5,\;\;\;\;\;\;\mu_{4}<Y_{WCW}^{*}, \end{array}$$

(3) $$\;Y_{WCB}=\{\begin{array}{c} \;\;1,\;\;\;\;\;\;\;\;\;Y_{WCB}^{*}<\lambda_{1}\\ 2,\;\;\;\;\;\;\;\;\;\lambda_{1}<Y_{WCB}^{*}<\lambda_{2}\\ 3,\;\;\;\;\;\;\;\;\;\lambda_{2}<Y_{WCB}^{*}, \end{array}$$

(4) $$\;Y_{DWB}=\{\begin{array}{c} \;-1,\;\;\;\;\;\;\;Y_{DWB}^{*}<\sigma_{1}\\ 0,\;\;\;\;\;\;\;\sigma_{1}<Y_{DWB}^{*}<\sigma_{2}\\ \;\;1,\;\;\;\;\;\;\;\sigma_{2}<Y_{DWB}^{*}. \end{array}$$

In the above three equations, $Y_{WCW},\;Y_{WCB},$ and $Y_{DWB}$ are ordered variables defined as 1–5 or 1–3, and $\mu_{i}$, $\lambda_{i}$ and $\sigma_{i}$ are the cut-off points of $Y_{i}^{*}$.

### 4.3. Descriptive Statistics

In Table 3, statistics show that 97.2% of the respondents are willing to participate in domestic waste classification, of which 82.3% present a high willingness and 48.1% report a very high willingness. However, only 88.5% of the respondents completely or partially sorted their waste in practice. Residents who comply with the four-type DWC standard required by Beijing’s policy only occupy 33.4% of the total. They simultaneously separate food waste, recyclables, and hazardous waste from other waste. Of respondents, only 16.9% separate food waste, whereas only 15.2% of residents separate both food waste and recyclables from the remaining waste. Of the respondents, 11.6% did not intentionally separate their waste, indicating that they did not actually participate in DWC.

The behavior of residents’ waste classification was improved after the implementation of the new round of DWC policies in Beijing, compared with the 2011 Beijing pilot1, the 2015 Ningbo case [9], and Beijing before the implementation of the comprehensive classification policies in 2020 [1]. However, there is still a gap between residents’ WCW and WCB, with the proportion of the former being higher than the latter. For instance, 97.2% of respondents reported WCW versus 88.5% of them conducting waste separation behaviors. One unique feature is that the deviation revealed by dataset in this study is relatively small compared to previous studies (only 8.7%). The traditional large deviation between WCW and WCB has been significantly narrowed down in Beijing. Therefore, it can be concluded that since the implementation of the Beijing Decree on Domestic Waste Management in 2020, the waste classification policy itself has been comprehensively recognized and followed by the public, and the practical waste separation behavior has been significantly enhanced. Meanwhile, 54.3% for respondents’ degree of waste classification willingness is higher than the standard degree of behavior. This phenomenon shows that existing policy measures have not promoted a full transformation of residents’ high willingness into faithful four-category-based separation behavior. Thus, there is still great potential for improvement in residents’ behavior toward a comprehensive and full-scale waste classification.

## 5. Empirical Results and Discussion

### 5.1. Regression on WCW and WCB

Based on the ordered logit model using robustness standard errors to address possible heterogeneities, the results of model (1) have been estimated with WCW or WCB as the dependent variables respectively (shown in Table 4). The valid sample size of the regression was 514 due to some missing data concerning the variables. The sign symbols of the variables showed an obvious consistency using WCW or WCB as the dependent variable, respectively. Among the five categories of influencing factors, the regression results showed satisfactory statistical significance, and the key determinants of WCW and WCB were identified in the context of Beijing. All analyses were performed using Stata 16.0 (StataCorp, College Station, TX, USA).

Among the attitude factors, satisfaction with property enterprise services is the key variable. It presents a significant positive correlation with residents’ WCW (0.223 **) and a significant negative link with residents’ WCB (−0.216 **). The main reason for this difference is that the higher the quality of daily property enterprise services, the more residents are willing to cooperate and support their waste management work to demonstrate a better performance of WCW by the residents. However, because property enterprises are in charge of waste classification management in communities, the secondary sorting in the front of public garbage bins has to be conducted to avoid punishment by township governments. In this case, residents’ incomplete classification, or even no classification, will not affect the actual effectiveness of DWC in the community as a whole, and the standard degree of WCB within households will be at a relatively low level. The results of the study also showed that residents’ environmental consciousness is positively related to WCW but had no significant effect on WCB. Knowledge of waste sorting and trust in classified transportation and disposal of domestic waste were positively associated with WCB, but had no significant effect on WCW, partially supported by the findings of Kuang and Lin [1] and Chen et al. [9].

Among the subjective norms factors, both the positive and negative waste classification behaviors of peers were negatively correlated with the WCW (−0.329 *, −0.887 ***) and WCB (−0.484 ***, −0.889 ***) of residents. Both legal and illegal separation behaviors reduced the degree of WCW and the standard degree of WCB. For the respondents, others’ positive classification behavior did not form a demonstration effect. In the case of the absence of regulatory measures, residents have a tendency to adopt a free-riding strategy to expect positive behaviors from others instead of themselves when conducting waste classification. Furthermore, residents’ psychological conformity is more negatively affected by others’ negative WCB, indicating that without effective supervision for DWC, the residential pattern of non-acquaintance society reduces the positive influence of subjective norms. It is a strong supplement to previous studies that have only examined the single positive effect of others’ behaviors [14].

Among the perceptual behavioral control factors, time cost is significantly negatively related to residents’ WCW (−0.019 **), but the significance with WCB did not pass the statistical test. Thus, it is empirically proven that the longer the time spent on DWC, the lower the residents’ performance on WCW. However, in real life, residents do not pay much attention to the actual time when sorting and putting out their waste, so there is no effect on WCB. Unlike the significant positive effect shown by the convenience of facilities in previous studies [9,22], the facilities convenience regarding waste classification as perceived by residents was at an appropriate level due to the considerable improvement in facilities such as government-provided trash bins in communities to promote residents’ DWC as a habit. In the communities investigated by this study, public garbage placed in each floor of the building has been withdrawn to prevent mixed waste throw. New four-category garbage cans have been stationed in the neighborhoods with sufficient numbers and reasonable location particularly since the beginning of the mandatory domestic waste classification phase in Beijing. So, there is no significant effect of facilities convenience on WCB.

Among the policy implementation effectiveness factors, the effectiveness of the existing fixed charge policy introduced before the comprehensive DWC pilot in Beijing (0.354 *) and the overall effect of community waste classification after the pilot (0.438 ***) played positive roles in promoting residents’ classification behavior, which is consistent with the conclusion of Miliute-Plepiene et al. [25]. But the two factors exerted no significant effect on WCW. Besides, the impact of other waste classification policies and measures in the communities on residents’ WCW and WCB failed to pass the significance test, indicating that the specific effect of current regulatory measures, economic incentive measures, and publicity measures is limited and needs to be further improved and strengthened.

Among the factors related to sociodemographic characteristics, WCW and WCB were significantly higher among women than men, and higher among older residents than younger ones. WCW was stronger among Communist Party of China (CPC) members but lower among high-income residents. Educational background had no significant effect on WCW or WCB.

### 5.2. Regression on Deviation between WCW and WCB

Based on the ordered logit model under robustness standard errors, the results of model (1) were estimated by directly using the deviation between WCW and WCB as the dependent variable. The results are shown in Table 4; several key findings can be revealed.

Among the attitude factors, both environmental consciousness (0.166 **) and satisfaction with property enterprise services (0.302 ***) were significantly positively correlated with the deviation between WCW and WCB. Both of them increased residents’ WCW, but the former had no effect on WCB, and the latter had a negative effect on WCB, thus widening the deviation.

Among the subjective norms factors, both positive WCB (0.302 ***) and negative WCB (0.468 **) of other residents were significantly positively correlated with the deviation between WCW and WCB of the residents themselves. The absolute value of the regression coefficients of both was greater than that of the willingness to sort. This means the negative effect on WCB was greater than that of WCW, and thus the deviation increased. This phenomenon also stems from the fact that the neighborhood living pattern of non-acquaintance society diluted the positive effect of subjective norms on garbage separation, and the residents’ mindset of free-riding on negative behaviors prevailed.

None of the perceptual behavioral control factors had a significant impact on residents’ deviation between WCW and WCB in this study. To some extent, it can reflect the reasonable layout and convenience of setting the four-type garbage public bins in terms of the number and location in the community.

Among the policy implementation effectiveness factors, the effectiveness of the existing flat charge policy of domestic waste before the comprehensive DWC pilot in Beijing (−0.332 *) and the overall effect of community waste classification after the pilot (−0.231 **) was negatively correlated with residents’ deviation between WCW and WCB. Publicity measures exerted a similar impact on the deviation (−0.159 **). The results mean that the three types of policy implementation effectiveness could regulate the WCB and promote a transformation from waste classification willingness to actual behavior, and then reduce the deviation. But the effectiveness of regulatory and incentives measures for deviation did not pass the significance test. The main reason for this may be that publicity measures are more intensive, including non-contact information and knowledge provision, community and property enterprises’ door-to-door agreements, distribution of relative materials, and fun activities on weekends. But with relatively low constraint forces, the potential for publicity measures to further reduce deviation in the future needs to be continuously examined and explored. Furthermore, regulatory and economic-incentive factors exerted a limited effect on enhancing residents’ waste-sorting behavior with respect to reducing willingness and behavior deviations. These factors included weak continuity with respect to supervision in front of garbage bins, the absence of penalties among regulatory measures, and that the low actual value of newly implemented incentive measures failed to provide sufficient economic incentives.

Among the sociodemographic characteristics, the deviation between WCW and WCB was greater for men than for women. The deviation existed across age and income classes and was not related to whether a resident is a CPC member.

Combining the results from Section 5.1 and Section 5.2, several conclusions can be drawn. First, except for environmental consciousness and gender, personal attitudes and sociodemographic characteristics such as knowledge, trust, time cost, political status, and income that play a role on WCW or WCB have no significant effect on the deviation between WCW and WCB. Therefore, deviation between WCW and WCB is sourced more from specific external conditions [21]. Second, attitudes and norms associated with subjects that are not authoritative regulators, such as the performance of property enterprises and neighboring residents, simultaneously influence residents’ WCW, WCB, and the deviations between the two. So, it is particularly important to optimize and properly deal with the relationship between residents and property enterprises during the implementation of DWC by installing suitable incentives and constraints to better motivate all residents to accomplish a thorough four-type waste classification required by DWC policies. Third, at present, Beijing’s DWC policies are mostly aimed at improving residents’ waste classification behaviors and thus reducing the deviations in willingness and behaviors. Although diversified publicity measures are not significant in promoting WCW or WCB alone, this measure can still achieve the goal of reducing the deviation. But with relatively low constraint forces, the potential of publicity measures to further reduce the deviation in the future needs to be continuously examined and explored.

### 5.3. Robustness Checks

This section checks the robustness of the baseline estimation results. First, ordered discrete dependent variables are applicable to both logit and probit models. Therefore, the results were re-estimated using the ordered probit regression method to check whether the results were consistent. The results in Table 5 indicate that the sign symbol and significance of the variables are almost identical to those in Table 4. The empirical results are robust to different estimation methods.

Considering that personal income information is more sensitive for individuals to provide, 17% of the surveyed respondents did not respond to this question. Additionally, the sign of parameter on income variable in the baseline regression did not match expectations. Thus, the income variable was removed to further test the robustness of the results. Table 6 shows that removing the income variable substantially increased the total sample observation from 514 to 621. And the sign symbol of the variable was almost unchanged (The change in sample size can cause some differences in the significance of some variables. Another concern is the possible high correlation between variables such as “Understanding of payment of fixed waste fee (UPF)” and income, The changes also indicate that policy making of DWC needs to pay attention to the attitude of different income groups), and the change in significance did not have a large impact on the main findings. The findings are typically robust.

## 6. Discussion

The main findings have been obtained in this paper. First, there is a deviation between the waste classification willingness and behavior of Beijing residents. Although the deviation in the binary classification case is moderate compared with that before the universal waste classification pilot in Beijing [1], 54.3% of respondents reported a higher degree of WCW than that of WCB, and only 33.4% of respondents can translate their willingness to the four-category complete classification required by the city government of Beijing. This result further illustrates that higher WCW does not necessarily mean more normative WCB. Second, there are differences in the determinants of WCW and WCB. Environmental consciousness plays a greater role in willingness, whereas knowledge and trust play a larger role in behavior. In contrast, the higher the satisfaction with property enterprise services, the better the performance of the residents’ WCW but the lower the WCB. The fixed waste charge policy introduced in 1999 and implementation effectiveness of the overall effect of community waste classification can improve residents’ WCB, but it had no significant effect on WCW. In the horizontal comparison of the influencing factors of WCW and WCB, the conclusions obtained from the newly added policy factors and property enterprise subject factors are complementary to the previous research conclusions [1,9,21]. Third, through the innovative deviation variables we constructed, the determinants of deviation between WCW and WCB is further explained. The deviation between WCW and WCB is associated with factors related to the residents themselves, neighboring residents, the services of property enterprises, and the regulations of the government, but more depends on specific external conditions [15], including attitudes and norms associated with other subjects that are not authoritative regulators, and the implementation of various policy instruments for DWC. The higher the satisfaction with property enterprise services, the higher the deviation between residents’ WCW and WCB. Moreover, both the positive and negative WCB of community peers increase the deviation. The effectiveness of government policies and measures can effectively reduce the deviation. Therefore, the impacts of external pressures brought about by other stakeholders’ behavior and attitudes also needs to be paid attention to in the study of factors influencing residents’ willingness and behavior.

The findings of this paper have significant policy implications. First, the finding of the role played by property enterprise subjects in promoting residents’ WCW and WCB, reveals that the government can consider synergistic cooperation with property enterprise in the implementation of DWC policies at least in the Chinese region. It is necessary to optimize and update the relationship between residents and property enterprises to implement proper incentives and constraints to motivate all residents to meet the standard of complete four-type waste classification through a combination of various government policies on domestic waste classification. Second, the existing fixed waste removal and disposal fees for residents can increase residents’ attention to DWC and thus reduce the deviation between residents’ WCW and WCB. However, the charging rates, which have lasted for more than 20 years, are extremely low, and the marginal cost of household garbage disposal under the fixed charge is zero. This kind of charge pattern lacks the economic incentives to reduce and classify domestic waste within households. Therefore, implementing a unit charging model to provide economic incentives for waste sorting and reduction is recommended. Third, from the perspective of economic rationality, residents will not internalize environmental externalities autonomously. Currently, there is no substantive penalty for classification violation. It is difficult to form sufficient subjective norms from peers’ behavior in the neighborhood. The phenomenon of “free-riding” has emerged. Therefore, it is necessary to improve public supervision and punishment policies. Several measures can be taken to reduce residents’ psychological conformity and to eliminate the collective action dilemma by matching regulatory measures with expected unit charge policy, strengthening the frequency of spot checks, and increasing the standard of violation fines. Fourth, a variety of publicity measures have shown their effectiveness in narrowing the deviation between WCW and WCB. So, it is proposed to further expand the publicity steps by enriching the content of publicity and education in order to enhance residents’ understanding of the meaning of DWC to facilitate accurate sorting operations. Several measures can be taken to further reduce the deviation between WCW and WCB, such as publicizing the unit charge policy as a supporting policy for DWC and increasing residents’ attention to DWC and environmental sanitation, so that environment-friendly information acquired by residents can be translated into intrinsic awareness and further a positive change in residents’ real behaviors.

With the dynamic changes of domestic waste classification policies, there are differences of the waste classification situation faced by residents in each city. The policy mix and specific policy measures adopted by each city for waste management are also be different. Since Beijing is still in the active implementation phase of the comprehensive domestic waste classification policy in 2021, the limited role of regulation and incentives in promoting waste classification willingness, implementing waste classification behavior, and reducing deviations between WCW and WCB shown in this paper is not necessarily indicative of the effectiveness of regulatory and incentive policies in other cities and in Beijing after that. Nevertheless, it still shows that the regulation and incentives for waste classification in Beijing need to be optimized based on the current situations.

## 7. Conclusions

This paper investigates the status and determinants of residents’ waste classification willingness, behavior, and the deviations between them. According to the extended framework of the theory planned behavior, this study analyzes five types of factors and 18 variables, including attitudes, subjective norms, perceived behavioral control, policy implementation effectiveness, and sociodemographic characteristics. In particular, after the full implementation of waste classification policy in Beijing, the effect of key policies on residents’ WCW, WCB, and their deviation is systematically evaluated. Significant deviation between WCW and WCB was revealed, which is determined by specific external conditions, including attitudes and norms associated with other subjects that are not authoritative regulators, and the implementation of various policy instruments for DWC. The flat charge policy for waste, community publicity measures, and the effect of community waste classification can facilitate a reduction in the deviation. However, the weak and unsustainable supervision of direct regulatory measures and ineffective economic incentives have played a limited role in promoting DWC. Therefore, this paper proposes to expand publicity and education means, consolidate residents’ knowledge of waste separation, shift the flat charge to a unit fee policy, and increase the frequency of spot checks and the amount of fines for illegality so that a larger part of WCW can be transformed into WCB, and residents’ free-riding behavior can be reduced to eliminate a collective action dilemma. The investigation has relevant practical implications for source DWC of residents in Beijing and even in other cities across China. For future research on DWC of urban residents, related research can be carried out by targeting additional cities to further reveal and analyze whether the deviation between WCW and WCB still exists. Among others, cities with diverse levels of economic development and social governance capacity deserve to be included and emphasized to compare the impact of income levels, different regulatory, incentive, and publicity measures on residents’ WCW, WCB and their deviation in different cities.

## Figures and Tables

**Table 1:** Basic characteristics of the questionnaire sample.

Characteristics	Category	Percentage (%)
Gender	Male	45.57
	Female	54.43
Age	below 18	0.63
	18–30	8.54
	31–45	37.82
	46–60	29.91
	Over 60	23.10
Education background	Primary school or below	1.11
	Junior high school	5.22
	High school/technical secondary school	13.29
	Associate college	18.67
	Bachelor	40.51
	Master’s degree and above	21.20
Political Status	Communist Party of China member	38.13
	Others	61.87
Personal annual income	Less than 30,000 yuan	13.96
	(30,000–100,000] yuan	46.27
	(100,000–300,000] yuan	34.23
	(300,000–500,000] yuan	4.02
	Above 500,000 yuan	1.53

**Table 2:** The definitions and descriptive statistics of the variables.

**Category**	**Variables**	**Symbol**	**Description**	**Min**	**Max**	**Mean**	**Sd.**
Dependent variables							
	Waste classification willingness	WCW	Willingness to waste classification (1 = very reluctant, 2 = relatively reluctant, 3 = neutral, 4 = relatively willing, 5 = very willing)	1	5	4.27	0.84
	Waste classification behavior	WCB	Behavior of waste classification (1 = no classification, 2 = partial classification (Separate partial waste of the following situations: only hazardous waste, only recyclables, only food waste, only recyclables and hazardous waste, only food waste and hazardous waste, or only food waste and recyclables), 3 = complete classification of food waste, recyclables, and hazardous waste)	1	3	2.22	0.63
	Deviation between WCW and WCB	DWB	Deviation between WCW and WCB (−1 = degree of WCW lower than that of WCB, 0 = degree of WCW equal to that of WCB, 1 = degree of WCW higher than that of WCB)	−1	1	0.50	0.58
Independent variable							
Attitude	Environmental consciousness	EC	Current urban waste pollution is serious. (1 = absolutely disagree, 2 = disagree, 3 = neutral, 4 = agree, 5 = completely agree)	1	5	3.54	1.20
	Knowledge of waste classification	KWC	I know a lot about DWC. (1 = absolutely disagree, 2 = disagree, 3 = neutral, 4 = agree, 5 = completely agree)	1	5	3.45	1.38
	Trust in classified transportation and disposal of waste	Trust	I believe that the separated waste will be classified transportation and disposed of properly. (1 = absolutely disagree, 2 = disagree, 3 = neutral, 4 = agree, 5 = completely agree)	1	5	3.72	1.42
	Satisfaction with property enterprise services	SPS	Satisfaction with property enterprise services (1 = very dissatisfied, 2 = relatively dissatisfied, 3 = neutral, 4 = relatively satisfied, 5 = very satisfied)	1	5	2.99	1.10
Subjective norms	Other residents’ positive WCB	OPB	Other residents’ classification will promote my sorting (0 = no, 1 = yes)	0	1	0.48	0.50
	Other residents’ negative WCB	ONB	I won’t sort waste if other residents don’t sort (0 = no, 1 = yes)	0	1	0.17	0.38
Perceptual behavioral control	Time cost	TC	Time taken by the current WCB or the expected time taken by the complete standard waste classification (numerical variable)	0	200	8.33	12.30
	Facilities convenience	FC	Current number and location of waste classification facilities in the community are reasonable (1 = absolutely disagree, 2 = disagree, 3 = neutral, 4 = agree, 5 = completely agree)	1	5	3.85	1.36
Policy implementation effectiveness	Understanding of payment of fixed waste fee	UPF	Payment of waste fixed waste fee (0 = don’t know, 1 = know)	0	1	0.32	0.47
	Overall effect of community waste classification	OE	Current DWC effectiveness in the neighborhood (1 = very poor, 2 = poor, 3 = fair, 4 = good, 5 = very good)	1	5	3.06	1.02
	Regulatory measures	RM	Regulatory measures taken in community known or accepted (four items can be checked, the variable conversion takes the value of 0/1/2/3/4)	0	4	0.72	0.81
	Incentive measures	IM	Incentive measures taken in community known or accepted (three items can be checked, the variable conversion takes the value of 0/1/2/3)	0	3	0.20	0.55
	Publicity measures	PM	Publicity measures taken in community known or accepted (seven items can be checked, the variable conversion takes the value of 0/1/2/3/4/5/6/7)	0	7	1.97	1.32
Sociodemographic characteristics	Gender	Gender	Dummy variable (0 = female, 1 = male)	0	1	0.46	0.50
	Age	Age	1 = below 18, 2 = 18–30, 3 = 31−45, 4 = 46−60, 5 = over 60	1	5	3.66	0.95
	Education background	Edu	1 = Primary school or below, 2 = Junior high school, 3 = High school/technical secondary school, 4 = Associate College, 5 = Bachelor, 6 = Master’s degree and above	1	6	4.56	1.18
	Political status	PS	1 = Communist Party of China (CPC) member, 0 = Other	0	1	0.38	0.49
	Income	Income	Personal annual income in 2020 (numerical variable)	0	800	14.51	36.52

**Table 3:** Description statistics of willingness, behavior, and deviation from DWC.

	Category	Detailed Description	Percentage (%)	Percentage (%)	Value
WCW	High willingness	Very willing	48.10	82.28	5
		Relatively willing	34.18		4
	Medium willingness	Neutral	14.87	14.87	3
	Low willingness	Relatively reluctant	2.22	2.85	2
		Very reluctant	0.63		1
WCB	Complete classification	Separate out food waste, recyclables, and hazardous waste	33.39	33.39	3
	Partial classification	Only separate out food waste and recyclables	15.19	55.06	2
		Only separate out food waste and hazardous waste	7.59		
		Only separate out recyclables and hazardous waste	4.27		
		Only separate out food waste	16.93		
		Only separate out recyclables	6.96		
		Only separate out hazardous waste	4.11		
	No classification	No intentional separation behavior	11.55	11.55	1
DWB	Degree of WCW higher than that of WCB	High willingness × No classification	7.91	54.27	1
		High willingness × Partial classification	43.51		
		Medium willingness × No classification	2.85		
	Degree of WCW equal to that of WCB	High willingness × Complete classification	30.85	41.61	0
		Medium willingness × Partial classification	9.97		
		Low willingness × No classification	0.79		
	Degree of WCW lower than that of WCB	Medium willingness × Complete classification	2.06	4.11	−1
		Low willingness × Partial classification	1.58		
		Low willingness × Complete classification	0.47		

**Table 4:** The results of ordered logit model.

	WCW	WCB	DWB
EC	0.257 ***	0.014	0.166 **
	(0.089)	(0.082)	(0.084)
KWC	0.066	0.127 *	−0.0972
	(0.077)	(0.075)	(0.079)
Trust	0.140	0.150 *	−0.037
	(0.089)	(0.084)	(0.083)
SPS	0.223 **	−0.216 **	0.302 ***
	(0.098)	(0.093)	(0.097)
OPB	−0.329 *	−0.484 ***	0.468 **
	(0.187)	(0.179)	(0.194)
ONB	−0.887 ***	−0.889 ***	0.496 *
	(0.218)	(0.236)	(0.273)
TC	−0.019 **	−0.002	−0.009
	(0.009)	(0.005)	(0.010)
FC	−0.035	−0.0114	0.0622
	(0.094)	(0.087)	(0.085)
UPF	0.127	0.354 *	−0.332 *
	(0.203)	(0.212)	(0.200)
OE	0.041	0.438 ***	−0.231 **
	(0.119)	(0.118)	(0.117)
RM	0.105	−0.139	0.116
	(0.138)	(0.136)	(0.141)
IM	0.077	−0.018	0.029
	(0.186)	(0.185)	(0.212)
PM	0.053	0.048	−0.159 **
	(0.086)	(0.077)	(0.078)
Gender	−0.329 *	-0.553 ***	0.432 **
	(0.180)	(0.185)	(0.187)
Age	0.392 ***	0.291 ***	−0.128
	(0.113)	(0.109)	(0.122)
Edu	0.008	−0.034	−0.021
	(0.090)	(0.091)	(0.095)
PS	0.362 *	0.342	0.075
	(0.209)	(0.208)	(0.229)
Income	−0.002 *	0.002	−0.001
	(0.001)	(0.002)	(0.001)
N	514	514	514
R2_p	0.0739	0.0828	0.0618

Notes: (1) Robust standard errors in parentheses; (2) * *p* < 0.1, ** *p* < 0.05, *** *p* < 0.01.

**Table 5:** The empirical results of ordered probit model.

	WCW	WCB	DWB
EC	0.151 ***	0.009	0.094 *
	(0.049)	(0.046)	(0.048)
KWC	0.027	0.075 *	−0.0972
	(0.044)	(0.042)	(0.046)
Trust	0.090 *	0.093 **	−0.001
	(0.050)	(0.047)	(0.050)
SPS	0.136 **	−0.127 **	0.188 ***
	(0.055)	(0.053)	(0.058)
OPB	−0.165	−0.294 ***	0.244 *
	(0.106)	(0.103)	(0.114)
ONB	−0.498 ***	−0.481 ***	0.240
	(0.123)	(0.132)	(0.161)
TC	−0.010 **	−0.001	−0.005
	(0.004)	(0.003)	(0.005)
FC	−0.012	−0.013	0.037
	(0.052)	(0.048)	(0.051)
UPF	0.113	0.157	−0.145
	(0.114)	(0.121)	(0.117)
OE	0.038	0.254 ***	−0.123 *
	(0.070)	(0.065)	(0.070)
RM	0.044	−0.079	0.064
	(0.084)	(0.077)	(0.084)
IM	0.029	0.006	−0.028
	(0.107)	(0.103)	(0.123)
PM	0.009	0.032	−0.096 **
	(0.050)	(0.045)	(0.045)
Gender	−0.187 *	−0.333 ***	0.246 **
	(0.105)	(0.107)	(0.109)
Age	0.227 ***	0.162 ***	−0.048
	(0.064)	(0.062)	(0.074)
Edu	0.009	−0.023	0.014
	(0.053)	(0.052)	(0.057)
PS	0.200 *	0.204 *	0.017
	(0.121)	(0.120)	(0.135)
Income	−0.001	0.001	−0.0004
	(0.001)	(0.001)	(0.001)
N	514	514	514
R2_p	0.0716	0.0810	0.0561

Notes: (1) Robust standard errors in parentheses; (2) * *p* < 0.1, ** *p* < 0.05, *** *p* < 0.01; (3) Yellow background indicates decreased significance, green background indicates increased significance, and the red color indicates symbol change.

**Table 6:** The results of ordered logit model without income variable.

	WCW	WCB	DWB
EC	0.160 **	−0.036	0.177 **
	(0.077)	(0.074)	(0.075)
KWC	0.100	0.161 **	−0.101
	(0.070)	(0.069)	(0.070)
Trust	0.123	0.094	0.001
	(0.083)	(0.079)	(0.076)
SPS	0.109	−0.161 *	0.182 **
	(0.088)	(0.082)	(0.086)
OPB	−0.100	−0.418 **	0.537 ***
	(0.166)	(0.164)	(0.176)
ONB	−0.855 ***	−0.883 ***	0.363
	(0.198)	(0.221)	(0.238)
TC	−0.014	0.002	−0.011
	(0.012)	(0.006)	(0.008)
FC	−0.055	0.014	0.030
	(0.087)	(0.080)	(0.080)
UPF	0.002	0.195	−0.259
	(0.181)	(0.200)	(0.182)
OE	0.150	0.349 ***	−0.075
	(0.106)	(0.109)	(0.106)
RM	0.025	−0.099	0.035
	(0.132)	(0.129)	(0.130)
IM	−0.003	0.078	−0.086
	(0.161)	(0.167)	(0.182)
PM	0.102	0.060	−0.135 *
	(0.077)	(0.072)	(0.073)
Gender	−0.344 **	−0.612 ***	0.477 ***
	(0.161)	(0.167)	(0.168)
Age	0.383 ***	0.254 **	−0.063
	(0.103)	(0.100)	(0.110)
Edu	0.020	−0.049	0.035
	(0.082)	(0.083)	(0.086)
PS	0.167	0.399 **	−0.103
	(0.184)	(0.191)	(0.199)
N	621	621	621
R2_p	0.0544	0.0690	0.0474

Notes: (1) Robust standard errors in parentheses; (2) * *p* < 0.1, ** *p* < 0.05, *** *p* < 0.01; (3) Yellow background indicates decreased significance, green background indicates increased significance, and the red color indicates symbol change.

## Data Availability

All the data are obtained from the random face-to-face survey of 632 households in Beijing and the Press conferences on the implementation of the 2020 Beijing Decree on Domestic Waste Management.
